# Vitiligo progression in a patient undergoing romosozumab treatment for osteoporosis

**DOI:** 10.1016/j.jdcr.2023.09.033

**Published:** 2023-10-11

**Authors:** Nicole Trepanowski, Rebecca M. Yim, Rachel Wetstone, Elizabeth MacDonald, Sarah Servattalab, Subin Jacob-George, John E. Harris

**Affiliations:** a Boston University School of Medicine, Boston, Massachusetts; b Department of Dermatology, University of Massachusetts Chan Medical School, Worcester, Massachusetts; c Tulane University School of Medicine, Tulane, Louisiana; d Florida International University College of Medicine, Herbert Wertheim College of Medicine, Miami, Florida

**Keywords:** adverse effect, antibody, BMP6, bone morphogenetic protein 6, Dynein light chain Tctex-type 3, Dynlt3, flare, injection, low-density lipoprotein receptor-related protein 5, low-density lipoprotein receptor-related protein 6, LRP5, LRP6, medication, Monoclonal, osteoporosis, Pathway, progression, activity, RANKL, receptor activator of nuclear factor κB ligand, Romosozumab, sclerostin, Signaling, vitiligo, Wnt, ß-catenin

## Introduction

Vitiligo is an autoimmune disease that results in skin depigmentation due to selective loss of melanocytes.^1^ The pathophysiology of vitiligo involves genetics, environment, autoimmunity, metabolic factors, oxidative stress, and cell attachment abnormalities.^1^^,^^2^ Downregulation of the Wnt/ß-catenin pathway and decreased ß-catenin have been reported in vitiligo lesions, leading some researchers to advocate treatment of vitiligo with Wnt/ß-catenin activators.^1^^,^^3^ Bone morphogenetic protein 6 (BMP6) stimulates melanogenesis and melanosome transfer from melanocytes to keratinocytes, and sclerostin is an antagonist of BMP6 in the skin.^4^ Romosozumab (Evenity, Amgen and UCB) is a sclerostin-neutralizing antibody; thus, it inhibits an inhibitor of the pathway, thereby promoting activation of Wnt/ß-catenin signaling.^5^ Herein, we present a case of a patient who experienced vitiligo progression while undergoing treatment with romosozumab for osteoporosis.

## Case report

A 66-year-old postmenopausal woman with a past medical history of osteoporosis, vitamin D deficiency, Raynaud phenomenon, and nonsegmental vitiligo presented with newly progressive depigmentation of the upper portion of the arms and back. The patient was recently diagnosed with osteoporosis and started receiving romosozumab 9 months prior, 210 mg administered at a clinic monthly (105 mg at each back side of the upper portion of the arm), with the last dose given the day before presentation. The patient noted that her previously stable vitiligo began spreading 1 to 2 months after starting romosozumab, most actively on the upper portion of the arms. The patient did not report other symptoms from the injections and was otherwise healthy. The patient’s family history was notable for type I diabetes mellitus in 2 first-degree relatives. Laboratory examinations were unremarkable, including normal thyroid function.

The patient noted the first depigmented spot on the face in her mid-30s shortly after giving birth. Within several years, the patient noted new areas of involvement on the hands, arms, legs, back, and feet. Many years prior, the patient had received treatment for the facial vitiligo with tacrolimus 0.1% ointment for 6 months, without clinical improvement. The patient had not pursued further treatment. Before presentation, the patient had been off treatment and with stable disease for years.

**Fig 1:**
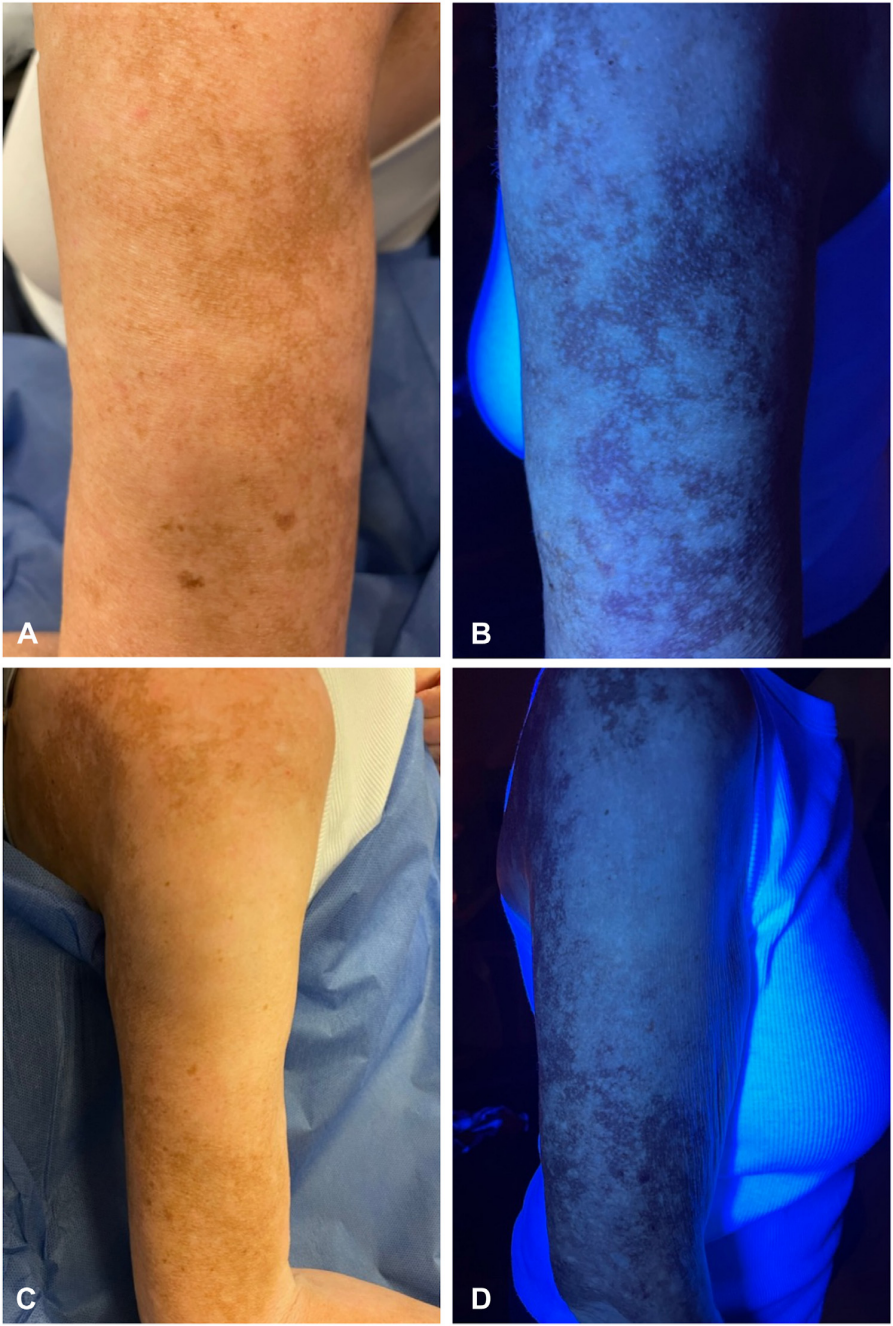
Natural light and Wood’s lamp examination of (**A, B**) the upper portion of the left arm and (**C, D**) upper portion of the right arm revealing extensive symmetric well-demarcated depigmented macules and patches with confetti-like macules.

Physical examination was notable for extensive symmetric well-demarcated depigmented macules and patches affecting approximately 75% of the body surface area (BSA), including the face, upper extremities, chest, abdomen, neck, back, lower extremities, hands, and feet. The face was 90% affected. Chalky white fluorescence on Wood’s lamp examination confirmed the diagnosis of vitiligo. Trichrome vitiligo was noted on the back, and confetti-like depigmentation was noted on the upper portion of the arms and back, suggesting active disease (Fig 1).^6^ Given the high BSA involvement, the patient is considering complete depigmentation with monobenzone 20% cream.

## Discussion

Romosozumab is a humanized monoclonal sclerostin-neutralizing antibody that binds to and inhibits sclerostin.^5^ It was approved by the US Food and Drug Administration in 2019 for the treatment of osteoporosis in postmenopausal women with a high fracture risk.^5^ Sclerostin is an osteocyte-derived glycoprotein that binds to low-density lipoprotein receptor-related proteins 5 and 6 (LRP5 and LRP6). This blocks activation of the Wnt/ß-catenin pathway and promotes the ubiquitinated degradation of ß-catenin. Sclerostin also increases the production of receptor activator of nuclear factor κB ligand (RANKL) and decreases the production of osteoprotegerin (OPG).^5^

Romosozumab inhibits sclerostin’s ability to bind to LRP5 and LRP6, promoting activation of the Wnt/ß-catenin pathway.^5^ This promotes osteogenesis and, to a lesser extent, inhibits bone resorption.^5^ Commonly reported side effects (in >5% of subjects) include arthralgia and headache.^5^ Injection site reactions, nasopharyngitis, back pain, erythema multiforme, dermatitis, and angioedema have also been reported.^5^

The Wnt/ß-catenin pathway has been linked to vitiligo pathophysiology.^1^ Studies have reported downregulation of the Wnt/ß-catenin pathway and reduced ß-catenin expression in vitiligo lesions.^1^ Upregulation of the Wnt/ß-catenin pathway might protect melanocytes from oxidative stress, inhibit CD8^+^ T cell differentiation into effector cells, and enhance regulatory T cells.^1^ The Wnt/ß-catenin pathway also promotes repigmentation by promoting the differentiation of melanocyte stem cells into melanocytes.^1^^,^^3^ Therefore, activation of the Wnt/ß-catenin pathway may halt progression of active vitiligo and promote repigmentation.^1^ Reported therapies for vitiligo, such as narrow-band UVB, 308-nm excimer laser, and vitamin D, are thought to activate Wnt/ß-catenin signaling^1^; however, there is contradictory evidence regarding ß-catenin activation and repigmentation. In melanocytes, activated ß-catenin decreases Dynein light chain Tctex-type 3 (Dynlt3) levels, reducing melanosome transfer to keratinocytes.^1^

**Fig 2:**
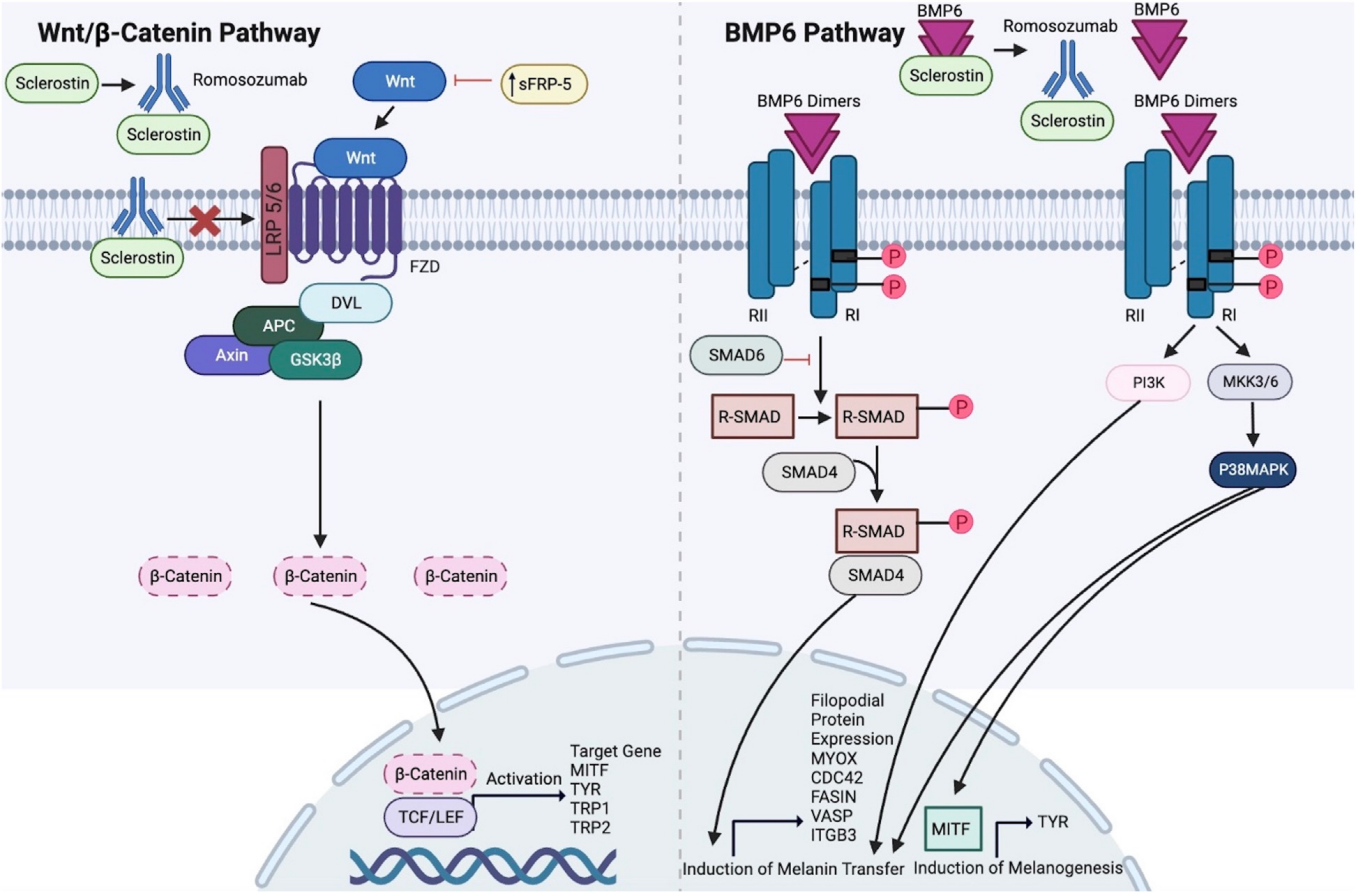
Proposed mechanism of action of romosozumab on melanocytes through the Wnt/ß-catenin and bone morphogenetic protein 6 pathways. Adapted from Zou et al, Lim et al, and Singh et al.^2^^,^^4^^,^^8^ Created with BioRender.com.

Strong sclerostin expression has been detected in both epidermal keratinocytes and melanocytes in healthy, normal-appearing skin.^4^ Sclerostin is an antagonist of BMP6, which stimulates melanogenesis by upregulating tyrosinase expression and activity as well as melanosome transfer from melanocytes to keratinocytes.^4^ Thus, inhibiting sclerostin could theoretically promote melanogenesis and repigmentation through BMP6. Interestingly, inhibiting sclerostin results in uveal melanoma progression through activation of the Wnt/ß-catenin pathway via binding of LRP5 and LRP6.^7^ These studies support that romosozumab, a sclerostin-neutralizing antibody, may have unintended effects on the skin. Fig 2 illustrates possible mechanisms of action of romosozumab on melanocytes.^2^^,^^4^^,^^8^

This case is medically interesting for multiple reasons. The patient’s newly active and progressive vitiligo after initiation of romosozumab suggest a correlation. The back sides of the upper arms, which were the medication injection sites, showed signs of disease activity (ie, confetti-like macules).^6^ As romosozumab activates the Wnt/ß-catenin pathway, romosozumab could theoretically halt vitiligo progression and promote repigmentation.^1^ Additionally, if romosozumab activates the BMP6 pathway by inhibiting sclerostin, this would further support that the medication promotes melanogenesis and repigmentation.^4^

However, in our case, we observed increased vitiligo activity after the use of romosozumab. The reason for this is unclear and requires further studies. It is possible that depigmentation at injection sites represents the Koebner phenomenon, but this would not explain the rapidly progressing vitiligo lesions on other sites (ie, the back).^9^ Alternatively, we may have observed a paradoxical reaction, an uncommon occurrence in which treatment with biologics intended to reverse disease can actually induce the disease.^10^ Although the correlation may be simply coincidental, our report suggests clinicians carefully consider the treatment of patients with vitiligo who are receiving romosozumab and potentially other Wnt pathway activators. Additionally, our findings question the hypothesis that Wnt pathway activators are beneficial to treat vitiligo.^1^^,^^3^ Further research is needed to investigate the relationship between romosozumab and vitiligo disease progression.

## Conflicts of interest

Dr Harris has the following conflicts of interest: 3^rd^ Rock Venture – Consultant (Fees); AbbVie, Inc – Consultant (Fees); Aclaris Therapeutics, Inc – Consultant (Fees), Investigator (Grants/Research Funding); Admirx – Consultant (Fees); Aldena – Consultant (Fees), Founder (Stock); Almiral – Consultant (Fees); AnaptysBio – Consultant (Fees); Avita – Consultant (Fees); BiologicsMD – Consultant (Fees); Boston Pharma – Consultant (Fees); BridgeBio – Consultant (Fees); Celgene – Investigator (Grants/Research Funding); Cogen Therapeutics – Consultant (Fees); Dermavant – Consultant (Fees), Investigator (Grants/Research Funding); Dermira – Consultant (Fees); EMD Serono – Consultant (Fees), Investigator (Grants/Research Funding); Frazier Management – Consultant (Fees); Genzyme/Sanofi – Consultant (Fees), Investigator (Grants/Research Funding); Granular Therapeutics, Inc – Consultant (Fees); Incyte – Consulting (Fees), Investigator (Grants/Research Funding), Equity; Janssen – Consultant (Fees); LEO Pharma – Consultant (Fees), Investigator (Grants/Research Funding); Matchpoint Therapeutics – Consultant (Fees); Merck – Consultant (Fees); NIRA Biosciences – Consultant (Fees), Founder (Stock); Pandion – Consultant (Fees); Pfizer – Consultant (Fees), Investigator (Grants/Research Funding); Platelet Biogenesis – Consultant (Fees); Rheos Medicines – Consultant (Fees), Investigator (Grants/Research Funding), Equity (Stock); Sonoma Biotherapeutics – Consultant (Fees); Steifel/GSK – Investigator (Grants/Research Funding); Sun Pharmaceuticals – Consultant (Fees), Investigator (Grants/Research Funding); Temprian Therapeutics – Consultant (Fees); TeVido BioDevices – Consultant (Fees), Investigator (Grants/Research Funding), Equity (Stock); Twi Biotech – Consultant (Fees); Villaris Therapeutics – Consulting (Fees), Investigator (Grants/Research Funding), Founder (Stock); Vimela Therapeutics – Consultant (Fees), Founder (Stock); Villaris Therapeutics was recently acquired by Incyte. All other authors have no conflicts of interest to disclose.
